# Miniaturized Folded-Slot CubeSat MIMO Antenna Design with Pattern Diversity

**DOI:** 10.3390/s22207855

**Published:** 2022-10-16

**Authors:** Rifaqat Hussain, Khaled Aljaloud, Abida Shaheen Rao, Abdullah M. AlGarni, Ali H. Alqahtani, Abdul Aziz, Yosef T. Aladadi, Saad I. Alhuwaimel, Niamat Hussain

**Affiliations:** 1 Electrical Engineering Department, King Fahd University for Petroleum and Minerals (KFUPM), Dhahran 31261, Saudi Arabia; 2 College of Engineering, Muzahimiyah Branch, King Saud University, Riyadh 11451, Saudi Arabia; 3 The Faculty of Engineering, The Islamia University of Bahawalpur, Bahawalpur 63100, Pakistan; 4 Department of Electrical Engineering, King Saud University, Riyadh 11421, Saudi Arabia; 5 King Abdulaziz City for Science and Technology, Riyadh 12354, Saudi Arabia; 6 Smart Device Engineering, Sejong University, Seoul 05006, Korea

**Keywords:** CubeSats, wide-band, folded slot, UHF band, reactive loading

## Abstract

In this paper, a folded slot-based multiple-input–multiple-output (MIMO) antenna design for Cube Satellite (CubeSat) applications is presented for the ultra-high frequency (UHF) band. A unique combination of a reactively loaded meandered slot with a folded structure is presented to achieve the antenna’s miniaturization. The proposed antenna is able to operate over a wide frequency band from 430~510 MHz. Moreover, pattern diversity is achieved by the antenna’s element placement, resulting in good MIMO diversity performance. The four elements are placed on one Unit (1U) for CubeSat dimensions of 100 mm × 100 mm × 100 mm. The miniaturized antenna design with pattern diversity over a wide operating band is well suited for small satellite applications, particularly CubeSats in the UHF band.

## 1. Introduction

Small satellites, such as Cube Satellites (CubeSats), have sparked a lot of interest in recent years because they enable quick and affordable platforms for technological demonstration, scientific study, and educational space programs. A CubeSat standard 1U size is 100 mm × 100 mm. The 1U may be easily upgraded to be used in bigger missions (2U to 12U). All of the basic operations of a conventional satellite, including altitude monitoring and control, uplink and downlink communications, and electrical power supply, are carried out in a CubeSat as well. Its energy needs are met by a battery unit and solar panels, which are installed on the body of the CubeSat. A CubeSat may be equipped with deployable solar panels and antennas. However, as CubeSats are limited in size as compared to conventional satellites, designing compact subsystems represents a challenging requirement for CubeSats applications. 

Antenna design is an important component in satellite systems for both the upstream and downstream communications that connect ground stations to satellites. Their size and weight should be in accordance with the CubeSat, and should provide good radiation performance [1]. In recent time, the requirements for Multi-Input–Multi-Output (MIMO) antennas for CubeSats operating at 437 MHz, i.e., the amateur UHF band, have increased. This has opened the doors for convenient uplink and downlink communication as well as convenient communications between different CubeSats in a network. Moreover, a planar MIMO antenna can play a key role in enhancing system throughput and diversity gain for CubeSat MIMO communication systems.

Suitable antenna designs for CubeSats in the UHF band, including both planar and non-planar designs, have been reported in the literature [2]. The various types of antennas include slot, dipole, monopole, helical, Yagi–Uda and meander-line antennas. However, planar antennas that are readily integrable with other RF and microwave circuits are gaining popularity. Planar antenna structures are suitable for utilizing the limited space available on a CubeSat for solar cells, which minimizes the likelihood of deployment failure. Various Patch antennas with planar geometry have been reported to operate in the UHF, L, S, C, and X bands. However, the large size of such antennas is a major drawback.

Various methodologies and approaches have been proposed to achieve a planar configuration with reduced antenna size [3,4,5,6,7,8,9,10,11,12,13,14,15,16,17,18,19]. However, simultaneously achieving miniaturization with a planar configuration, wide bandwidth, and higher gain, particularly in the UHF band, remains a challenging task. Slot antennas are considered a suitable option to achieve the required planar configuration for CubeSat communication applications. However, most of the proposed slot antenna designs for CubeSats in the UHF band suffer from the disadvantages of linear polarization and low directivity.

The patch antenna design proposed in [3] has a fractal structure and a small size of 60 mm × 26.3 mm; however, it has low gain. The design proposed in [5] examined a dual resonant antenna and a miniaturized slot antenna with a size of 5.73 cm × 5.94 cm × 0.05 cm. It showed a gain of 1.7 dB and good impedance matching. In [6], a miniaturized slot antenna with size of 5.5 cm × 5.5 cm × 0.787 cm at 0.3 GHz was presented. A slot line with an electrical length of λ/4 was shorted with an inductor. The main observed drawback of this design is its reduced bandwidth of 1.6% (4.8 MHz). The slot antenna proposed in [7] has a size of 5.327 cm × 5.327 cm × 0.05 cm. The authors improved its bandwidth and efficiency by increasing the folded slot aperture over the slot proposed in [6] at the cost of reduced gain, although with better bandwidth and radiation efficiency.

Two slot-based cavity-backed antennas for UHF uplink and downlink communication were demonstrated in [8] by a loop meandered line formed by wrapping and mounting a circularly polarized slot around the CubeSat. The antennas operate at 485 MHz (UHF) and 500 MHz (UHF), respectively, and both demonstrated the same gain of 4 dBi at both frequencies. 

In [9], two antennas were proposed, one a folded structure and the other a self-complementary planar structure with a size of 10 cm × 8 cm × 0.0787 cm. The first antenna design consisted of a miniaturized folded printed wire and had a wide bandwidth of 0.60% (0.336 GHz). The second antenna design with a self-complementary H-shaped structure had a wider bandwidth of 2.1% (1.3 GHz). The slot antenna proposed in [10] had a high gain of 12.45 dB, however, it had narrow bandwidth and a larger size of 16 cm × 17 cm × 0.68 cm. In [11], another meandered-line antenna design was proposed in which nylon is used as a substrate; it operates at 437 MHz in the UHF band. The bandwidth and reflection coefficient obtained for the flat configuration was 5 percent and −22 dB, respectively. Its bent configuration has a total gain of 4.1 dBi, as compared to the flat one with 3.88 dBi.

In Table 1, a detailed comparison is made to compare the distinguishing features of the most relevant slot antennas for CubeSats at the UHF band available in the literature. The most important features for comparison are antenna size, antenna type, gain, bandwidth, and radiation efficiency. It can be seen that the proposed MIMO antenna achieved a higher size reduction of 66%, better radiation efficiency of 92%, and significantly better bandwidth performance. This confirms the appropriateness of the proposed meandered folded-slot antenna element with capacitive loading to achieve a miniaturized MIMO antenna with better radiation efficiency, bandwidth, and pattern diversity performance in the UHF band for CubeSat applications. The UHF band is suitable for long-distance communications, better penetration, and low losses. In addition, the frequency allocation for CubeSat links is regulated by international entities. Typically, the suitable frequency bands used for CubeSats are the very high frequency (VHF) and UHF bands.

This study presents a highly miniaturized folded slot-based MIMO antenna with pattern diversity. The four-element MIMO antenna is intended for 1U of the CubeSat surface, and has dimensions of 100 mm × 100 mm × 100 mm. Miniaturization is achieved in the proposed structure by folding a meandered slot line together with capacitive loading. The proposed antenna is distinguished by its planar geometry, wide-band operation, pattern diversity, and extremely down-sized antenna structure in MIMO configuration. Each folded slot is 50 mm × 68 mm in size and operates in a frequency range of 430–510 MHz with 80 MHz of bandwidth for CubeSat applications. To the best of our knowledge, the suggested folded slot-based MIMO antenna design with planar geometry is the first of its kind that is both highly miniaturized and able to demonstrate a wide bandwidth.

## 2. Proposed Folded-Slot CubeSat MIMO Antenna

### 2.1. Antenna Geometry

**Figure 1:**
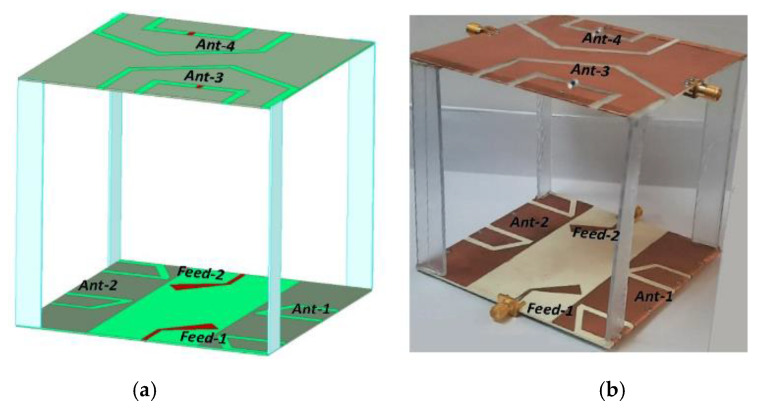
Proposed folded-slot CubeSat MIMO antenna: (**a**) simulated and (**b**) fabricated model.

Figure 1 shows the proposed miniaturized MIMO folded-slot antenna design operating in the UHF band with a single surface dimension of 100 mm × 100 mm. Rogers RO4350 substrate with dielectric constant of 3.48 and loss-tangent of 0.0036 was employed as a substrate material. This substrate was chosen due to its low loss and ease of milling with LPKF-103.

**Figure 2:**
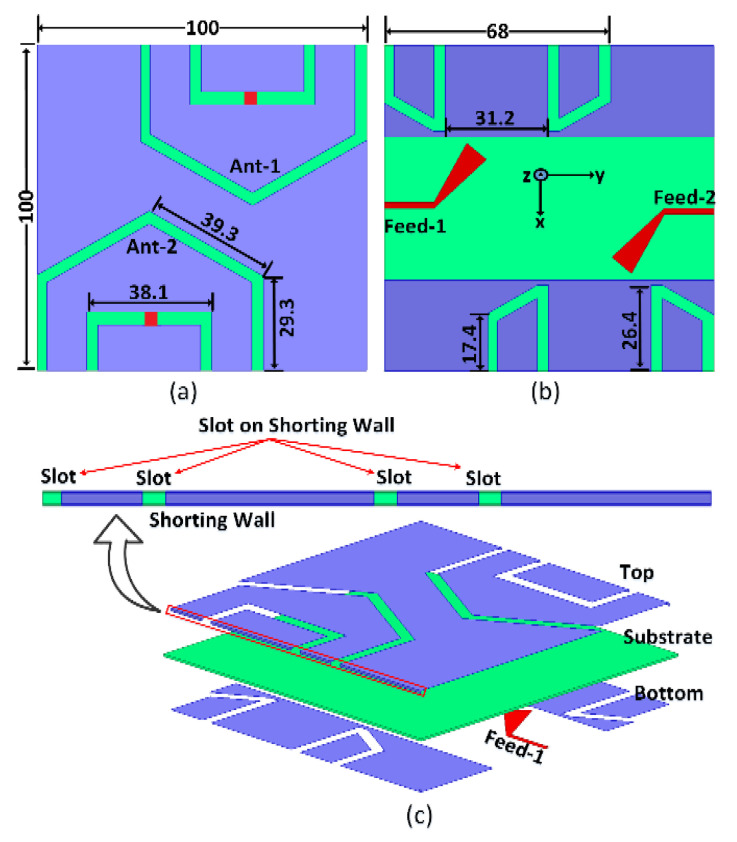
Proposed antenna: (**a**) top view, (**b**) bottom view, (**c**) detail of various antenna layers.

**Figure 3:**
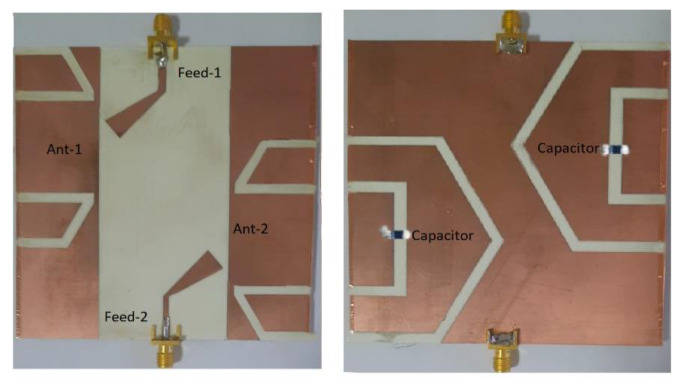
Fabricated prototype of the proposed CubeSat MIMO antenna: (**a**) top view and (**b**) bottom view.

Figure 2a,b illustrate the top and bottom layers of the MIMO antenna as well as the detailed antenna dimensions. Each slot is 2 mm in width. Figure 2c illustrates different antenna layers. The proposed antenna design was fabricated using LPKF S103, and is shown in Figure 3.

### 2.2. Antenna Design Procedure

The proposed meandered folded-slot antenna is designed to achieve an antenna with reduced sized, planar geometry, wideband operation, and pattern diversity in a MIMO configuration. The folding technique aided in the creation of a very compact antenna with extremely reduced size, allowing for the addition of a second antenna element in a MIMO configuration, as described below. The antenna’s top layer, as illustrated in Figure 2a, is comprised of two slot antennas. Ant-1 is shaped and curved in such a way that it can be folded down from the upper right corner. This allows enough room for Ant-2 to be placed on the bottom left cover of the top layer, with its slot folded in the same way as Ant-1. Ant-1 occupies half of the square area of the supplied substrate board, as can be observed from the antenna topology. The same procedure is repeated for Ant-3 and Ant-4. The bottom layer shown in Figure 2b is comprised of feed lines (Feed-1 and Feed-2) as well as the remaining section of the folded slots. The Ant-1 and Ant-2 antenna elements are excited by Feed-1 and Feed-2, respectively. The layers of the MIMO antenna are depicted in Figure 2c. By extending the slot to the opposite side of the substrate, the slot on the shorting wall can be used to extend the electrical length. The antennas’ dimensions are greatly reduced by their folded structure. To further reduce antenna size, the slot can be reactively loaded with a capacitor. In this way, the novel feeding mechanism and combination of folded structure with capacitive loading help to achieve a compact MIMO antenna with pattern diversity in the UHF band.

## 3. Simulation and Measurement

The MIMO antenna was designed, optimized, and analyzed using Ansys Electromagnetics Suite. The proposed antenna was then fabricated and measured to verify the simulated results. The parameters of the folded slot elements and feed structure along with the capacitance values were carefully tuned to achieve a compact four-element MIMO antenna able to cover the 430–510 MHz frequency band with greater than 10 dB return loss. The optimized values of all the structure parameters are provided in Figure 2, with the capacitance value of 8 pF adjusted for the optimized design. The tuning capacitor was chosen by parametric analysis. We carried out several iterations to arrive at the final optimized value of the tuning capacitor. The frequency band of the antenna may be controlled in order to shift further towards lower frequencies or towards higher frequencies by adjusting only the capacitance value. Hence, the proposed antenna design has the necessary adaptability and flexibility for use in other frequency bands. A fabricated prototype of the proposed MIMO antenna is shown in Figure 3a,b. The Agilent FeldFox network analyzer was used to characterize the prototype for S-parameters.

### 3.1. Scattering Parameters

**Figure 4:**
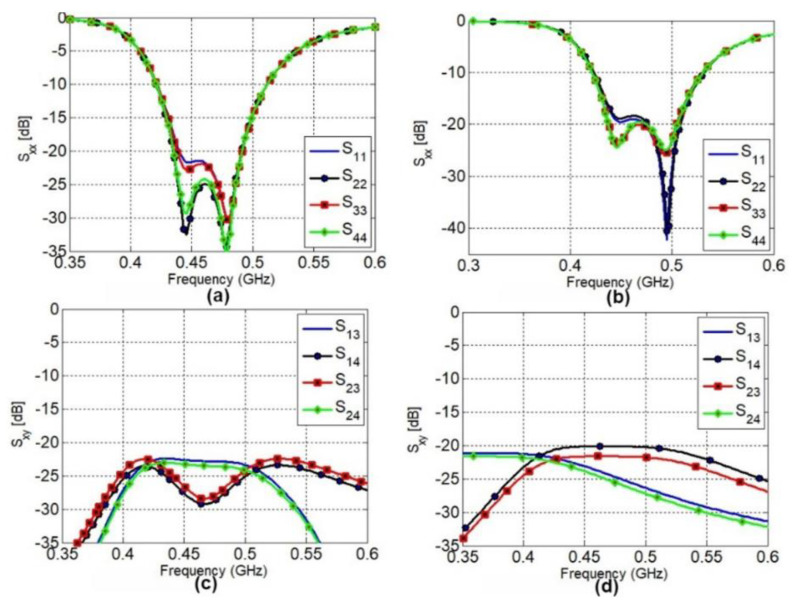
Proposed CubeSat MIMO antenna scattering parameters: (**a**) simulated *S_xx_* (**b**) measured *S_xx_* (**c**) simulated *S_xy_* (**d**) measured *S_xy_*.

Figure 4 illustrates the results of simulated and measured s-parameters for all of the four antenna elements. It can be seen that the measured and simulated scattering parameters are in good agreement. The reflection coefficients for all four antenna elements are well below −10 dB for simulated and measured results in the 430–510 MHz frequency band, as shown in Figure 4a,b, respectively.

Furthermore, both the simulated and measured transmission coefficients are below −20 dB in the mentioned frequency band, which are considered good isolation levels for a MIMO antenna. The majority of planar antennas designed for the UHF band described in the literature have a restricted bandwidth. However, the proposed MIMO antenna is able to achieve a −10 dB measured impedance bandwidth of at least 80 MHz.

### 3.2. Input Impedance

**Figure 5:**
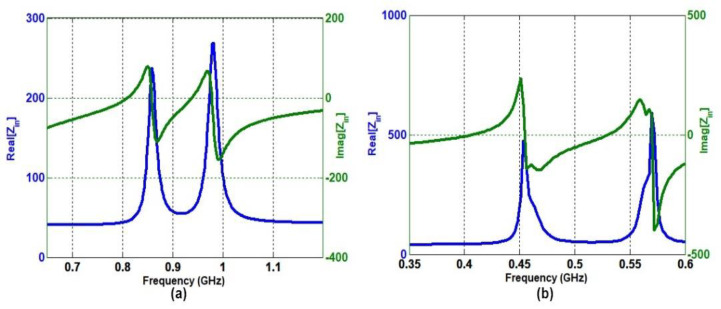
Proposed CubeSat MIMO antenna input impedance Z_in_ (**a**) without capacitor and (**b**) with capacitor.

The antenna was realized and tuned at the desired frequency band by reactive loading of the folded slot. Figure 5a,b show the input impedance curves for the MIMO antenna with and without capacitive loading, respectively. The real and imaginary values of Z_in_ at the resonance frequencies are real positive values around 50 Ω for ReZ_in_, while ImZ_in_ is nearly equal to zero. The capacitive loading helped in bringing down the resonance at the desired lower frequency band. Ideally, for resonance, both real and imaginary parts should be 50 Ω and zero, respectively. However, it is quite challenging to achieve both together. From Figure 5, it can be seen that either the real or the imaginary parts are close to ideal conditions, indicating the achievement of better resonance. The input impedance matching at lower frequency bands for electrically small antennas is quite challenging. The following steps helped us to better tune the antenna at a lower frequency band.

Several parametric sweeps were performed on the position of the slot from the edges. We found that keeping the antenna near the edge helped with better input impedance matching.

- Parametric analysis on the width of the slot;
- Position of the input feedline;
- Length of the input feed line

### 3.3. Radiation Characteristics

**Figure 6:**
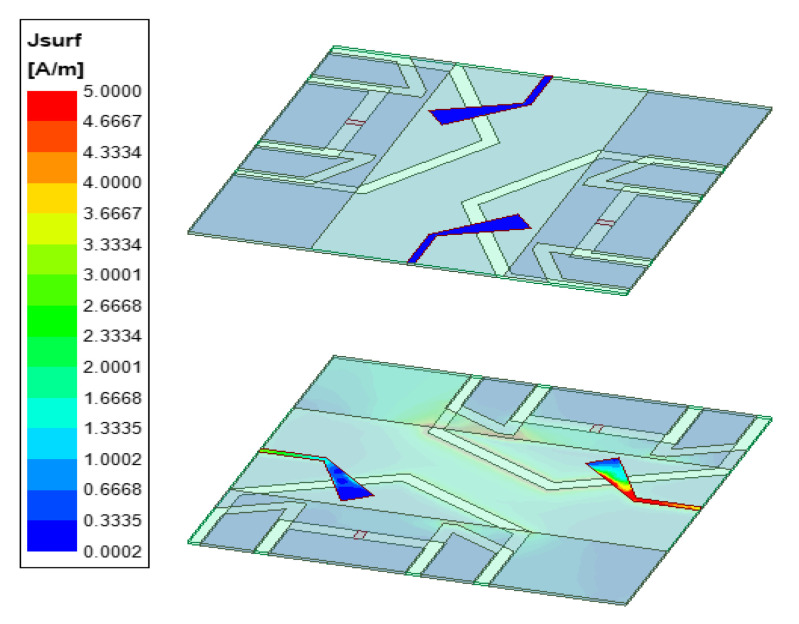
Fabricated antenna current density distributions (top and bottom view).

The proposed antenna design was characterized for current density distributions and far field patters. Figure 6 shows the current density distribution by exciting a single element at the lower substrate board. It can be seen that the other ports are well isolated.

**Figure 7:**
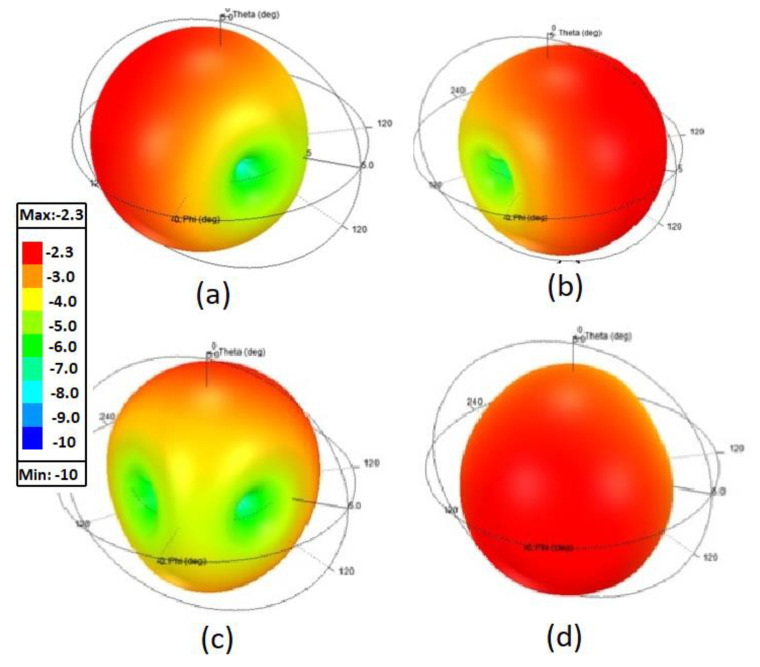
3D radiation patterns of the proposed CubeSat MIMO antenna at 450 MHz: (**a**) Ant-1, (**b**) Ant-2, (**c**) Ant-3, (**d**) Ant-4.

The far-field radiation patterns of the proposed folded-slot MIMO antenna were analyzed as well. The peak gain and radiation efficiency (%η) values for each antenna element were calculated, while other ports of the MIMO antenna elements were terminated with a 50 Ω load for each measurement. Figure 7 shows the 3D gain radiation patterns for all four antenna elements.

**Figure 8:**
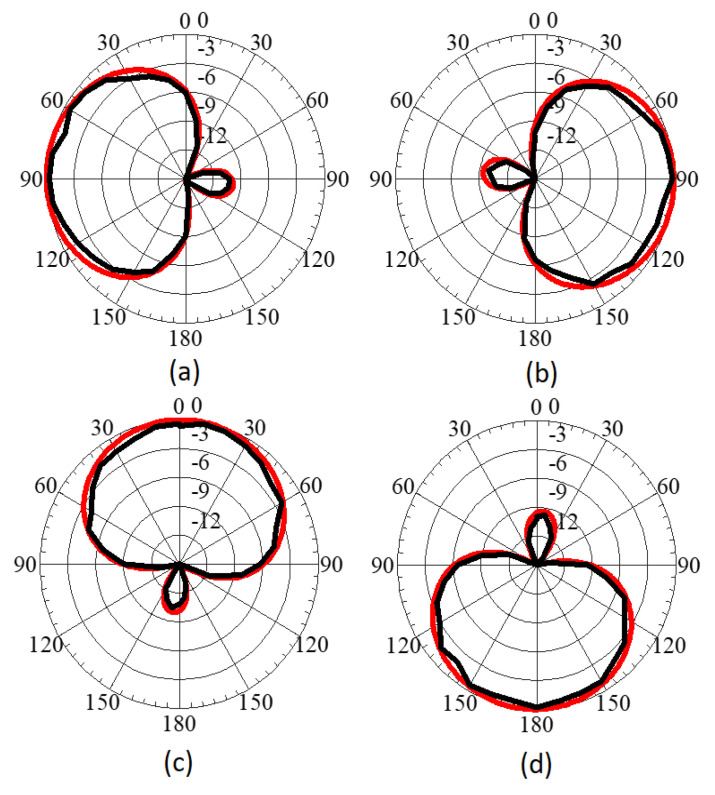
Simulated and measured radiation patterns of the proposed CubeSat MIMO antenna at 450 MHz: (**a**) Ant-1, (**b**) Ant-2, (**c**) Ant-3, (**d**) Ant-4. The red lines show simulated data, while the black lines denote measured data.

The antenna elements achieve a peak gain of −2.3 dBi at 435 MHz with 92% radiation efficiency. The simulated and measured radiation patterns of the proposed CubeSat MIMO antenna at 450 MHz are shown in Figure 8. The measurement data validate the numerically computed results. From Figure 7 and Figure 8, it can be seen that all the antenna elements radiate in orthogonal directions, which helps to reduce field coupling between the MIMO antenna elements, and hence in achieving pattern diversity. Such measures are helpful in enhancing the diversity gain performance of the proposed MIMO antenna. The proposed antenna is related to class of miniaturized antennas for CubeSat applications. In general, miniaturized antennas have low gain values, especially at lower frequency bands, due to the smaller electrical size of the antenna.

### 3.4. Envelope Correlation Coefficient

The envelop correlation coefficient (ECC) can be calculated using s-parameters and far-field results by solving Equations (1) and (2), respectively [20,21,22]:

(1) $$\rho_{eij}=\frac{{\left|S_{ii}*S_{ij}+S_{ji}*S_{jj}\right|}^{2}}{\left(1-{\left|S_{ii}\right|}^{2}-S_{ij}{}^{2}\right)\left(1-{\left|S_{ji}\right|}^{2}-S_{jj}{}^{2}\right)}\;$$

(2) $$\rho_{eij}=\frac{{\left|\iint_{0}^{4\pi }\left[\vec{R_{i}}\left(\theta ,\;\varphi \right)\times \vec{R_{j}}\left(\theta ,\;\varphi \right)\;\right]d\Omega \right|}^{2}}{\iint_{0}^{4\pi }{\left|\vec{R_{i}}\left(\theta ,\;\varphi \right)\right|}^{2}d\Omega \iint_{0}^{4\pi }{\left|\vec{R_{j}}\left(\theta ,\;\varphi \right)\right|}^{2}d\Omega \;}\;$$

**Figure 9:**
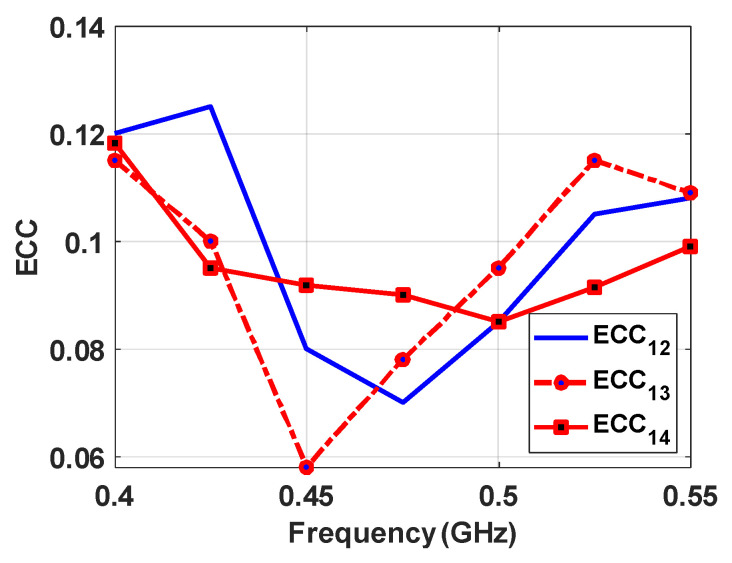
ECC between various antenna elements of the proposed CubeSat MIMO antenna.

To verify diversity gain performance and field coupling between various MIMO channels, ECC was computed using the far-field method for accuracy. The MIMO antenna performed well for ECC values due to the unique orientation of four-elements antennas, which resulted in orthogonal orientation for a maximum gain pattern in different directions. The ECC curves between various antenna elements are shown in Figure 9; it can be seen that the result is less than 0.12 over the entire operating frequency band. This confirms the satisfactory diversity performance of the proposed MIMO antenna.

## 4. Conclusions

A miniaturized MIMO antenna suitable for CubeSat applications is proposed for the UHF frequency band in this work. A novel combination of a meandered slot with a folded structure and capacitive loading is used to achieve a compact and miniaturized antenna design in the UHF band with pattern diversity. The folded slot is capacitively loaded for further reduction in antenna size and fine-tuning for the frequency band. The proposed antenna was operated in the 430–510 MHz frequency band and achieved a minimum −10 dB measured bandwidth of 80 MHz with good radiation efficiency and significant size reduction. The measured performance of the proposed MIMO antenna (far-field characteristics and diversity performance) confirms its potential for applications in CubeSat applications.

## Figures and Tables

**Table 1:** Comparison between the proposed CubeSat MIMO antenna and other works.

References	Size (λ_0_^2^)	Type of Antenna	Gain (dBi)	Freq. Bands (MHz)	Fractional BW (%)	%η
[5]	0.145 × 0.15	Double Slot	1.7	852	2.4	-
[6]	0.05 × 0.05	Slot	−3.0	300	-	20
[7]	0.067 × 0.067	Folded Slot	−2.7	338	0.9	35
[8]	0.32 × 0.26	Cavity Backed Slot	4.0	485/500	3.0	-
[9]	0.065 × 0.065	Self-Complementary Slot	−4.5	336	1.1	19
**This Work**	**0.075 × 0.102**	**Folded Slot**	**−2.3**	**437**	**17**	**92**

## Data Availability

Not applicable.
